# Validation of the inhaler adherence questionnaire

**DOI:** 10.1186/s40359-020-00461-x

**Published:** 2020-09-03

**Authors:** Brett G. Toelle, Guy B. Marks, Stewart M. Dunn

**Affiliations:** 1 Woolcock Institute of Medical Research, The University of Sydney, Camperdown, NSW Australia; 2 Sydney Local Health District, Camperdown, NSW Australia; 3 South Western Sydney Clinical School, University of New South Wales, Sydney, Australia; 4 Ingham Institute of Applied Medical Research, Sydney, Australia; 5 Department of Psychological Medicine, The University of Sydney, Camperdown, NSW Australia

**Keywords:** Adherence, Asthma, Compliance, Inhaler use, Questionnaire

## Abstract

**Background:**

Although electronic monitoring is the “gold standard” for adherence monitoring, the range of inhaler devices on the market exceeds the availability of appropriate monitoring devices. Simple tools, applicable across a range of inhalers, are needed to assess patients’ adherence to prescribed inhaled medication. This study reports on the validation of an Inhaler Adherence Questionnaire (IAQ).

**Methods:**

Seventy-four adults who self-reported doctor diagnosed asthma and who were prescribed daily inhaled corticosteroids (ICS) for asthma contributed data for these analyses. These participants were part of a larger study, investigating factors associated with non-adherence to prescribed daily inhaled corticosteroid medication. Participants were informed the research was investigating asthma management without explicit mention that medication adherence was being monitored. Inhaled corticosteroid medication adherence was measured in two ways. Firstly, participants completed the 6-item IAQ at enrolment. Secondly, ICS via pressurised Metered Dose Inhaler (pMDI) use was monitored electronically using the DoserCT which recorded daily use over 6 weeks. During the 6 weeks of prospective medication monitoring via the DoserCT we did not have contact with participants so that the adherence measure would reflect usual self-management behaviour.

**Results:**

Two of the six questions in the IAQ had poor face validity and their exclusion from the questionnaire resulted in improved internal consistency. Mean days adherent were 37.1, 29.2 and 33.2% for subjects with IAQ scores of 0, 1 and 2 respectively. Higher IAQ scores of 3 and 4 were associated with greater mean days adherent of 73.7 and 67.4% respectively. A cut-point of 2 or less had a sensitivity of 73% and a specificity of 80% for detecting non-adherence. The area under the ROC curve was 0.764 (*p* < 0.001).

**Conclusions:**

The modified 4-item IAQ is simple, quick to complete and useful for measuring adherence with prescribed daily inhaled medication. This validation of the IAQ provides evidence for its utility in research and it will be important to validate this simple, inexpensive tool for use in clinical practice.

## Background

Patient non-adherence with prescribed medication is common in all areas of medicine. Research has demonstrated that physicians have difficulty accurately identifying which of their patients are likely to be non-adherent [1–4]. Objective measurement of adherence has a role in assisting clinicians with management of patients who are failing to respond to existing treatment. In this situation valid information about non-adherence can avert inappropriate escalation of treatment in an attempt to obtain symptom control [5]. In the context of clinical trials, assessment of adherence is also important to enable identification, among people who volunteer as participants, of those who are likely to be adherent to the medication under investigation [6, 7].

Measuring adherence to inhaler therapy in people with asthma poses some specific challenges for clinicians and investigators. It has been suggested for decades that electronic medication monitors provide the best information about medication adherence for inhalers [8] and more recently in a systematic review of adherence and asthma exacerbations the authors wrote “Electronic device monitoring is usually considered the gold standard because of a detailed assessment of adherence patterns” [9]. However, the plethora of inhalation devices available [10], which includes the pressurised Metered Dose Inhaler (pMDI), Turbuhaler, Autohaler, Accuhaler, Rapihaler, Spiromax, Breezhaler, Respimat, Handihaler, Genuair and Ellipta devices, makes it difficult for developers of electronic monitoring devices to develop a feasible set of monitors. Therefore, it is important to have access to valid, non-device-specific instruments to measure adherence across the range of inhaler devices on the market. Questionnaire-based tools are a feasible option.

The Morisky Medication Adherence Scale [11] has been widely implemented as a medication adherence questionnaire and was adapted to develop the 6-item Inhaler Adherence Scale (IAS) [12]. Some preliminary evidence about its construct and discriminative validity and its reliability has been published [12, 13]. But, the lack of validation data for self-report adherence measures is not surprising and in a recent systematic review that included the IAS, the authors wrote “However, there is limited information about which types of scales are most acceptable and non-intrusive to patients, are the most reliable and obtain the most accurate information [14]. This is true for the IAS and its agreement with the reference standard, electronic monitoring, has not been evaluated. In light of the large range of inhaler devices available, the lack of electronic monitoring devices for these inhalers and the simplicity, low cost and ease of use of the Inhaler Adherence Scale we sought to validate the Inhaler Adherence Questionnaire (IAQ) against electronically monitored adherence to prescribed daily inhaled corticosteroid therapy.

## Methods

### Study participants

Participants representing a broad range of severity of asthma were recruited from two sources. The first source was adults who attended the outpatient specialist referral clinic of a large metropolitan teaching hospital. At their initial clinic visit, patients were asked to complete a questionnaire that collected information about their medical history, treatment and whether they consented to being contacted about research projects. The second source was a database of adults who had their name recorded on the research volunteer database at the Woolcock Institute of Medical Research. This database contains the names of adults who had participated in previous research studies and who agreed to be contacted in the future. It also contained the names of adults who had contacted the Institute in response to an advertisement or a media item and who had consented to be entered into the volunteer database.

Eligible participants were adults who self-reported both doctor diagnosed asthma and prescribed daily inhaled corticosteroid medication by pressurised metered dose inhaler (pMDI), and who agreed to participate in research and who were fluent in English. All participants provided informed written consent. The protocol was approved by the University of Sydney Human Ethics Committee (Ref 01/08/44).

### Study design

Potential participants were contacted by telephone or mail and informed that we were conducting research investigating the way people manage their asthma. Participants were told they would be sent questionnaires and a device that measures the dose of inhaler medicine used. All participant questions seeking further information or clarification were answered honestly and completely. However, we did not offer any mention of adherence or compliance. Participants who agreed to participate and who met the study criteria were enrolled and mailed the study materials with instructions to wait for our next telephone call to connect the supplied device to their inhaler. Seven days after the recruitment telephone call participants were again telephoned to ensure the package containing the adherence questionnaire and electronic monitor had arrived and to assist them to attach the electronic monitor to their prescribed daily corticosteroid inhaler. At this time, they also provided information about their asthma and asthma management and returned these study materials via mail.

The analyses presented here represent a sub-study within a larger study of factors associated with non-adherence to prescribed daily inhaled corticosteroid medication. As part of the larger study, participants also completed questionnaires that measured anxiety, depression, asthma knowledge, personality, perceived involvement in care, optimism, and asthma severity.

### Measurements of adherence

The Inhaler Adherence Questionnaire [13] is a 6-item Yes-No self-completed questionnaire that takes less than 1 min to complete. A yes is scored 0 and a no answer is scored 1 with a higher score indicating inhaler adherence.

The inhaler adherence electronic monitor we used was the DoserCT (Meditrack, Hudson, MA) [15] which attached to the canister of the corticosteroid pMDI and recorded the number of actuations per 24-h with sufficient memory for 45 days. The DoserCT has the added advantage of being able to operate in blind mode so that no actuation information is displayed by the device. Because the DoserCT was attached to the pMDI it did not matter whether the participant did or did not use a spacer. The Doser has been compared with the MDI Log (Medtrac Technologies; Lakewood, CO); the SmartMist (Aradigm Corporation; Hayward, CA) and the Nebulizer Chronolog (Medtrac Technologies; Lakewood, CO) and has been shown to be sufficiently accurate to monitor adherence [16, 17]. At enrolment participants recorded their prescribed inhaled corticosteroid regimen. During the 6 weeks of electronic monitoring participants made a diary entry only to record changes to their regimen either initiated by their doctor, as outlined in an asthma action plan or self-initiated. The DoserCT and diary were returned by mail.

### Electronically monitored adherence definition

Although there is no consensus definition of adherence, the definition used in this study combined the measurement capability of the DoserCT with results reported from previous studies. Electronically monitored adherence was measured using DoserCT data from days 8 to 42, to exclude days when the DoserCT was in transit and to discard any enrolment or learning effect [18]. Participants were classified as adherent if they used the prescribed number of actuations per day, or more, for at least 70% of days. The definition of adherence as 70% of days having used the prescribed or greater than prescribed dose of inhaled corticosteroid was based on previous research [18, 19] and our clinical experience. Pragmatically, selecting an adherence cut-off point of 70% allows for participants who regularly take their prescribed medication but miss the occasional dose, or who miss doses while waiting to get a prescription filled or who forget to take their medication with them when away from home to still be defined as adherent.

### Analyses

Participants in this study were drawn from a larger study assessing factors associated with non-adherence to prescribed daily inhaled corticosteroid medication. Assuming a non-adherence prevalence estimated of 50%, a minimum sample size of 40 participant were required to achieve a minimum power of 80% for detecting a change in the percentage value of sensitivity of a screening test from 0.50 to 0.80, based on a target significance level of 0.05. With the same assumptions, detecting a change in sensitivity of the screening test from 0.50 to 0.70 required a minimum sample size of 98 [20].

All data were analysed using the SAS statistical package for Windows [21]. Spearman’s rank order correlation is a non-parametric test used to assess both the direction and the strength of association between two variables [22]. The internal consistency of the IAQ was tested using Cronbach’s alpha [23]. Cronbach’s alpha reflects how closely a set of items in a questionnaire are related and can be interpreted as a measure of scale reliability. The validity of the IAQ as a measure of adherence was quantified as sensitivity and specificity in relation to the objectively measured adherence using the electronic monitor. Sensitivity and specificity were measured each cut point on the IAQ score and the area under the curve (AUC) was estimated. The area under the curve provides information about the diagnostic accuracy of the test. An AUC value of 0 reflects a perfectly inaccurate test and a value of 1 indicates a perfectly accurate test [24].

## Results

A total of 89 participants were recruited into this study and 74 (83%) participants provided both IAQ and DoserCT data for analyses. The 15 participants who did not return data did so for a variety of reasons including withdrawal of consent, study materials lost in the mail, medication changed to dry powder inhaler, materials and telephone calls not returned. A total of 6 DoserCT malfunctioned and did not have any recorded data when returned. In this case we supplied participants with a new DoserCT to their inhaler for a further 6 weeks and we were able to collect useable data from these participants.

Of the 74 study participants for whom complete data were available, 30 (41%) were recruited from the asthma clinic and 39 (53%) were male. With regard to asthma characteristics, 40 (56%) reported having wheeze at least each month, 56 (76%) reported having an asthma management plan and 50 (68%) had ever been admitted to hospital for asthma with 12 (25%) of these admissions occurring within the last 12 months. The median age was 52 (IQR 25) years and the median age at diagnosis of asthma was 12 (IQR 35) years.

Table 1 shows the IAQ questions, the proportion that answered yes and the Spearman correlation matrix. Questions four and six were not correlated with other questions in the IAQ. Cronbach’s Alpha was 0.73 with all six questions included and increased to 0.80 when questions four and six were removed. Further analyses were performed using only the four items of IAQ, so that the IAQ provided a total score between 0 and 4.

**Table 1:** Proportion of sample answering yes to each question and the Spearman correlation matrix for all six questionnaire items

Please think about your use of your [name of inhaled corticosteroid] In the last 3 months …	Proportion YES	Q1	Q2	Q3	Q4	Q5	Q6
Q1. … have you at times been careless about using your inhaler?	47.3	1.00	0.62*	0.48*	0.12	0.60*	0.26
Q2. … have you ever forgotten to use your inhaler?	59.5	0.62*	1.00	0.28***	0.10	0.40**	0.21
Q3. … have you ever stopped using your inhaler because you felt better?	28.4	0.48*	0.28***	1.00	0.19	0.64*	0.06
Q4. … have you ever stopped using your inhaler because you felt worse?	1.4	0.12	0.10	0.19	1.00	0.14	0.23**
Q5. … have you ever used your inhaler less than the doctor prescribed because you felt better?	40.5	0.60*	0.40**	0.64*	0.14	1.00	0.06
Q6. … have you ever used your inhaler more than the doctor prescribed because you felt you were having an attack?	20.3	0.26	0.21	0.06	0.23**	0.06	1.00

*p < 0.0001

**p < 0.005

***p < 0.05

Overall, 40.5% of the study population were classified as adherent because they used their inhaler as prescribed or more than prescribed on at least 70% of days on the basis of electronic monitoring.

**Fig. 1:**
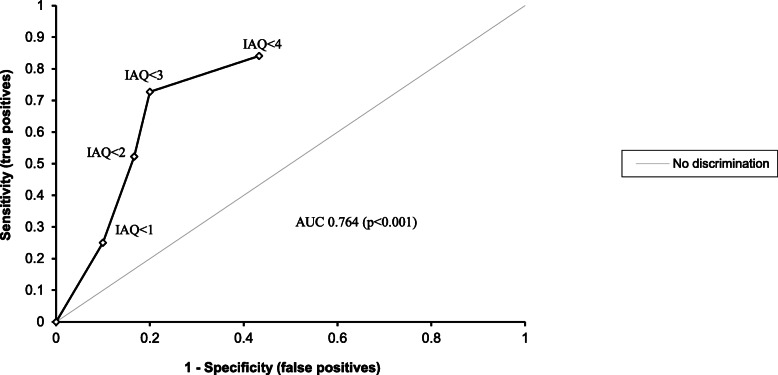
Receiver Operating Characteristic (ROC) curve for each score on the Inhaler Adherence Questionnaire predicting non-adherence measured by DoserCT

Figure 1 shows the receiver operating characteristic (ROC) curve for the IAQ predicting adherence. A cut-point of 2 or less had a sensitivity of 73% and a specificity of 80% for detecting non-adherence. The area under the ROC curve was 0.764 (*p* < 0.001). Comparison of the area under the ROC curve for adherence defined as 65% days, 75% of days and 80% of days adherent medication use were not different, providing evidence that the validity of the IAQ score was not dependent on the chosen electronically monitored adherence cut point.

## Discussion

The four-item IAQ is a simple, easy to use, internally consistent and valid tool to identify adult patients with asthma who are likely to be non-adherent with their daily prescribed pMDI corticosteroid regimen.

Non-adherence continues to be a major challenge in chronic disease management, despite many years of research into methods to measure adherence, identify risk factors and develop interventions. In fact, authors of a recent review wrote “Indeed, in the last decades, the degree of nonadherence remained unchanged”. They reported objectively measured ICS adherence from two studies in children that ranged from 20 to 33.9% and from three studies in adults ranged from 15 to 54% [25]. This suggests that there are still important questions to be answered to address medication non-adherence.

A limitation of this study may be that participants had all volunteered to be contacted about research and this might represent a particularly motivated sub-population of adults who have asthma. However, our objective in recruiting participants was to include those with a range of levels of adherence and this appears to have been achieved as analysis of electronically monitored adherence did confirm that participants in this validation study had a wide range of non-adherence to medication use. Similarly, the 83% completion rate and the variety of reasons for non-completion reassured us that no particular type of participant was more likely to withdraw.

Another limitation of this study is that it was possible for participants in this study to actuate their inhaler on days when they either took no or less than prescribed medication. This would have the effect of making them appear to be adherent with their medication regimen. However, we attempted to minimise this happening by referring to the study as investigating asthma management, not specifically referring to the ability of the DoserCT to measure medication adherence and after the initial contact not making any further participant contact until after the 6 week monitoring period.

The finding that questions 4 and 6 did not perform well is consistent with the findings of Brooks et al [13]. In their report they advocated for the retention of these questions to enable comparison with hypertension research. However, in our view the internal consistency and face validity of the IAQ was improved with the removal of both these questions and we recommend that these questions are not included and that future application only include the four-item IAQ for use in asthma.

The IAQ score derived from this simple questionnaire is a pragmatic tool for predicting adherence and non-adherence to prescribed daily inhaled corticosteroid therapy in research settings. We have shown that the use of these IAQ score improves the ability of any health professional to assess the likelihood of inhaler adherence and can be used in the research setting when it is not possible or feasible to collect electronically monitored adherence but when it is extremely important to have information about medication use to guide enrolment into a trial or to enable interpretation of study results. This validation study supports the future use of this tool in research and, while is likely that it will be useful in primary care, this still needs to be tested.

General practitioners and members of the health care team have long understood that non-adherence is a major barrier to optimal disease management. However, they themselves face a range of barriers to be able to satisfactorily address non-adherence. A recent qualitative study with General Practitioners identified four key areas including: patient-specific factors, the healthcare system, characteristics of drug therapies and the function and role of healthcare professionals as a team. In fact, the GPs who participated in this research identified interprofessional practice which takes a team approach as desirable and also asked for tools to enable the identification of non-adherence [26].

In standard clinical care, physicians can use this questionnaire as an aid to determine whether they should address barriers to adherence as a starting point or move more quickly towards a change in medication to obtain asthma control. Patient and physician interactions that incorporate good communication and acknowledge patient experience about barriers to adherence have been recommended and incorporated into numerous adherence interventions [25]. The responses to the actual questions in the IAQ provide an excellent starting point to discuss behavioural strategies and beliefs about medications to enhance adherence.

## Conclusions

This long overdue electronic validation of the IAQ has shown that this simple 4 item questionnaire is a valuable tool for assessing inhaler adherence, with application in the research and potentially in clinical settings. In today’s world where electronic devices, smartphones and the internet of things are promoted as solutions to all our problems, sometimes a return to the past can be beneficial. I’m reminded of the 1974 Peter Allen song “Everything Old is New Again”.

## Data Availability

The datasets used and/or analysed during the current study are available from the corresponding author on reasonable request.

## Abbreviations

IAQ: Inhaler Adherence Questionnaire
IAS: Inhaler Adherence Scale
ICS: Inhaled Corticosteroid
pMDI: Pressurised Metered Dose Inhaler
ROC: Receiver Operating Characteristic
AUC: Area Under the Curve
